# Eating Habits in Older Adults: Compliance with the Recommended Daily Intakes and Its Relationship with Sociodemographic Characteristics, Clinical Conditions, and Lifestyles

**DOI:** 10.3390/nu12020446

**Published:** 2020-02-11

**Authors:** Ana Zaragoza-Martí, Nicolás Ruiz-Robledillo, Miriam Sánchez-SanSegundo, Natalia Albaladejo-Blázquez, Jose Antonio Hurtado-Sánchez, Rosario Ferrer-Cascales

**Affiliations:** 1 Nursing Department, Health Psychology and Human Behavior Research Group, Faculty of Health Sciences, University of Alicante, 03690 Alicante, Spain; ana.zaragozaz@ua.es (A.Z.-M.); ja.hurtado@ua.es (J.A.H.-S.); 2 Department of Health Psychology, Health Psychology and Human Behavior Research Group, Faculty of Health Sciences, University of Alicante, 03690 Alicante, Spain; miriam.sanchez@ua.es (M.S.-S.); natalia.albaladejo@ua.es (N.A.-B.); rosario.ferrer@ua.es (R.F.-C.)

**Keywords:** nutrient intake, older adults, dietary recommendation, healthy ageing

## Abstract

Background: Older people have different nutritional requirements from those of the general population; in particular, they need a lower energy intake, higher protein content to preserve muscle mass, and a greater supply of vitamins and minerals to maintain good bone health. The objective of this study is to evaluate the degree of compliance with nutritional recommendations, and its relationship with sociodemographic characteristics, clinical conditions, and lifestyles in older people residing in the Spanish Mediterranean. Methods: Cross-sectional study with 341 people over 60 years old. Participants were selected using a snowball strategy. A validated food intake frequency questionnaire for older populations was used to determine the daily food intake. This evaluation was carried out at two time points from which the average nutrient intake was obtained. Sociodemographic, clinical, and lifestyle variables were obtained from an ad hoc elaborated questionnaire. Results: Compliance with dietary intakes was low, by deficiency, such as in vitamin D, where none of the participating subjects met the requirements, and iodine, where the compliance rate did not exceed 20%, or by excess, such as with monounsaturated fatty acids, fiber, iron, B vitamins, vitamin E, and vitamin C. People with better blood pressure, cholesterol, and glucose levels observed a higher degree of compliance with the recommended intakes. Living in rural areas, being divorced, or being illiterate negatively influence meeting the recommended intakes of certain nutrients. Increased physical activity was associated with an increased compliance with Kcal recommendations, cholesterol, and vitamin B2 intake. Conclusion: this study highlights the importance of accurately knowing the dietary intakes in the older population, and what factors, such as lifestyles or sociodemographic characteristics, may predispose to better or worse compliance with the recommendations.

## 1. Introduction

**Figure 1:**
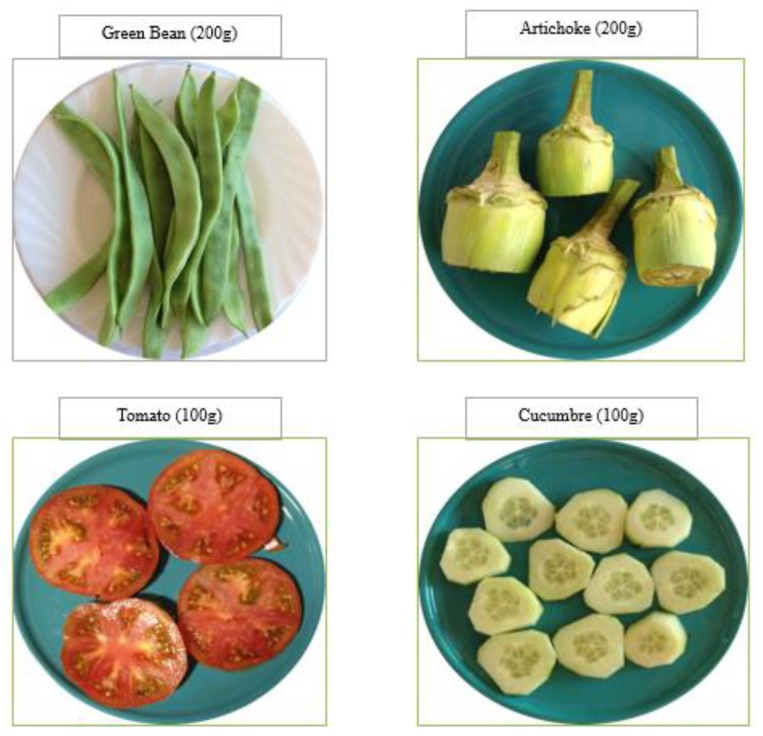
Example of the photo album.

The world’s population is ageing significantly, with an estimated 2.1 billion people being over the age of 60 in the year 2050, doubling the current figure [1]. In particular, in Spain, there was a total of 8,764,204 people over the age of 60 in 2017, representing 18.8% of the total population. According to the Instituto Nacional de Estadística (INE; National Statistics Institute), it is estimated that by 2066, there will be 14 million older people, 34.6% of the total population [2]. This demographic shift is a global change that can affect the economy, politics, work environment, and public health [3].

The aging process has been linked to a number of psychological changes, such as a deterioration of individuals’ cognitive and functional abilities [4,5], as well as the presence of a number of comorbidities, such as a loss of muscle mass, digestive problems, deteriorating oral health, malnutrition, fragility, and the development of chronic-degenerative diseases. All of this causes a loss of health-related quality of life [6]. All of these changes can be aggravated if an active lifestyle and healthy eating are not maintained, increasing the associated health costs [7].

Among all of the above-mentioned factors, nutrition is worth highlighting as a significant and easily modifiable risk factor for the prevention of various diseases [8]. Numerous research studies have shown that healthy nutrition is related to better health and higher health-related quality of life in older people [8,9,10,11,12]. Therefore, it is very important to know the dietary patterns of these people in order to detect and prevent nutritional deficits and/or excesses, and thus prevent the onset of certain age-related diseases. In addition to knowing the dietary patterns, it is necessary to know how these patterns are associated with sociodemographic factors, with the aim of designing specific strategies to promote health in this group according to their needs [13].

Older people have different nutritional requirements from those of the general population; in particular, they need a lower energy intake, higher protein content to preserve muscle mass, and a greater supply of vitamins and minerals to maintain good bone health [6]. Essential micro-nutrients such as vitamins, minerals, and trace elements, although needed in very small amounts, play a key role in functional maintenance, growth, and development throughout the life cycle. A deficiency or excess of any of these nutrients can cause serious alterations in the body both at the metabolic and the psychological level [14]. Older people may be at more risk of suffering this type of alteration, as the aging process can lead to a greater loss of essential nutrients, such as calcium, zinc iron, B vitamins, vitamin D, and high-quality biological proteins [15].

Such nutritional problems could be prevented and/or controlled through a healthy eating pattern, characterized by an increased consumption of vegetables, fruits, whole grains, legumes, and fish, and a low consumption of sweets, refined foods, and processed meats, as well as an active lifestyle. In this sense, it is very important for this population group to meet the specific nutritional requirements for its age and physiological status [16]. Recent studies have shown that those who do not meet the nutritional requirements have an increased risk of fragility, cardiovascular disease, osteoporosis, cachexia, malnutrition, and cognitive decline [17,18]. In this regard, some sociodemographic, clinical, and lifestyles factors could influence adherence to nutritional recommendations. For example, in a previous study conducted in Spain, it was demonstrated that some sociodemographic characteristic could modulate adherence to nutritional recommendations [19]. Similar results have also been obtained in other European regions, such as Switzerland [6] and Luxembourg [20]. According to clinical variables, another recent study conducted with 13,000 patients from France demonstrated that the clinical status of individuals could have a significant effect on dietary compliance [21]. Similarly, lifestyles could also have an influence, such as physical exercise [19] or a smoking habit [20].

On the other hand, the World Health Organization (WHO) estimates that with the elimination of risk factors associated with chronic-degenerative diseases, such as tobacco, alcohol, sedentary lifestyle, and poor food, the risk of cardiovascular disease, stroke, and type 2 diabetes could be reduced by 80% [22].

Therefore, the objective of this study is to assess the degree of compliance with nutritional recommendations and its relationship with sociodemographic characteristics, clinical conditions, and lifestyles in older citizens residing in the Spanish Mediterranean.

## 2. Methodology

### 2.1. Study Population

A cross-sectional study with a total of 341 people over 60 years old, residing in the Spanish Mediterranean. A total of 420 people were invited to participate in the study, from whom we received a response rate of 80%, so the final sample included a total of 341 people. All of them were volunteers and signed informed consent prior to their participation in the study. Participants were selected from an environment close to the interviewers, using a snowball strategy. All of the subjects who were dependent for basic daily life activities, with a score of three or more errors in Pfeiffer’s test, and those who could not read and write were excluded from the study. Furthermore, participants who were under dietetic intervention during the last year and those consumed some nutritional supplement were also excluded.

### 2.2. Ethical Considerations

This study was carried out in accordance with the fundamental principles set out in the Helsinki Declaration, as well as the requirements established in Spanish legislation in the field of biomedical research, data protection, and bioethics, and in accordance with the European Union’s Standards of Good Clinical Practice. This study was approved by the Ethics Committee of the University of Alicante (UA-2016-02-11). To protect the strict confidentiality of the data, codes were assigned to identify the participants in the study. Once the information was collected, a member of the research team downloaded the data onto a database. At no time was any personal information that could identify the participants included in the database. All of the participants read and signed the informed consent to participate in the study.

### 2.3. Instruments

#### 2.3.1. Dietary Intake: Food Consumption Frequency Questionnaire (MEDIS-FFQ)

The MEDIS-FFQ questionnaire was validated for the older population living in the Spanish Mediterranean, showing good psychometric properties, with a moderate–high Kappa index between 0.71 and 9.99 [23]. This questionnaire consisted of several food and beverage groups (11 groups), namely: dairy products, cereals and foods with starch, meat and meat products, fish, legumes and traditional dishes, vegetables, fruits and nuts, snacks (meat pies, etc.), sweets, drinks, and fats. The questionnaire also evaluated the size of the rations (small, medium, or large) and specified what type of bread (whole wheat or white) or what type of fat (olive oil, margarine, etc.), and what type of cheese or beverage was consumed. The frequency of consumption referred to the last year’s period, and the frequencies were daily consumption (once a day or more than twice a day), weekly consumption (once to twice weekly, and three to six times weekly), monthly consumption (one to three times a month), and no consumption or occasional consumption. In order to quantify seasonal food consumption (fruits and vegetables), participants were asked how often they consumed these foods during the season. In order to help participants quantify their actual food intake, a food photo dossier composed of all of the food consumption frequency questionnaire (FFQ) items was elaborated with the actual size of the rations (Figure 1). Food photo dossier available: https://www.dropbox.com/s/ilu839ulatpcp3z/Historial%20de%20fotos%20%20Nueva-nota-copyright-2.pdf?dl=0.

All of the consumption frequencies were recategorized into a single frequency in order to obtain the average daily intake of each food, and the nutrient intake was estimated using the food composition table of the Institute of Nutrition and Technology of Food from the University of Granada [24,25].

#### 2.3.2. Sociodemographic, Clinical, and Lifestyle Data

An ad hoc questionnaire was used to collect the sociodemographic, clinical, and lifestyle data. The sociodemographic data contemplated in our study were age, sex, marital status, years of schooling, and place of residence. The clinical variables studied were blood glucose levels, blood cholesterol levels, systolic and diastolic blood pressure, weight, and height. Lastly, the lifestyle variables studied were alcohol use, tobacco use, and physical activity (hours/weeks)

#### 2.3.3. Anthropometric Data

Standardized methods were used to measure the anthropometric data. Body weight was measured using a vertical mechanical scale with SECA 700 sliding weights, with an accuracy of 100 g. Height was measured with an accuracy of 0.2 cm, using the vertical statimeter. With the weight data in kg and height in cm, the body mass index (BMI = weight/height^2^; Kg/m^2^) was calculated. BMI was interpreted using the WHO’s classification (BMI < 18.8 = low weight, BMI between 18.5–24.99 = normal weight, BMI between 25–29.9 = overweight, and BMI > 30 = obesity).

### 2.4. Procedure

The measurements of the variables were performed by trained personnel with experience in assessing nutritional status and administering questionnaires. All of the questionnaires were completed on a computer by the interviewers at the same time of the interviews through an ad hoc elaborated booklet.

In order to increase the reliability of the intake assessment, the assessment of the frequency of the food intake was carried out over two time periods, with a nine-month interval between the two assessments, from which the ingestion of nutrients was obtained.

### 2.5. Analysis

Descriptive analyses were used to describe participants’ characteristics and mean intake of food, energy (Kcal), and nutrients. Most of the variables on food and nutrient intake followed a normal distribution, so, in this case, parametric tests were used.

The responses obtained from the FFQ on the frequency of consumption of each food were converted to daily frequencies, using the average value of each category. Coefficients of 0.0, 0.07 (2/30), 0.21 (1.5/7), 0.64 (4.5/7), 1.0, and 2.5 were used to indicate frequencies of never or almost never, one to three times a month, one to two times a week, three to six times a week, once a day, and two or more times a day. These coefficients were then multiplied by the food quantities of each item expressed in grams, thus obtaining the daily amount of grams consumed. The individual estimate of the daily and nutrient intake was calculated using Mataix Verdú’s food composition tables [22], adjusting by edible portion. The mean daily dietary intakes of energy, macronutrients, and micronutrients, as well as their distributions were compared with age- and gender-specific Spanish recommendation nutrients intake [26].

The chi-square test was used to determine the differences between participants who met the recommendations and those who did not comply with the recommendations. The Bonferroni test was used to determine significant differences between alcohol consumption, tobacco use, marital status, level of study, physical activity, BMI, and age.

The analyses were carried out with the SPSS statistical package, version 26 (IMB Corp, Alicante, Spain), and the level of statistical significance established for all of the tests was 0.05.

## 3. Results

### 3.1. Sociodemographic Characteristics

A total of 340 subjects with an average age of 70 years (standard deviation (SD) = 8.59), with 59.1% (*n =* 201) women and 40.9% (*n =* 139) men, participated in the study. Table 1 shows the sociodemographic variables and lifestyles of the participants.

### 3.2. Nutrient Intake

Table 2 shows the mean nutrient intake and its recommended value as a function of sex, for both sexes. The total caloric intake for women was 2522,08 Kcal, of which 15.80% was protein, 45.80% was lipids, and 36.23% was carbohydrates. In the case of men, the total caloric intake was 2330 Kcal, of which 15.70% was protein, 48.28% was lipids, and 33.65% was carbohydrates.

We observed an excess in the intake of proteins; lipids; monounsaturated and polyunsaturated fatty acids; fiber; iron; and vitamins B1, B2, B6, B12, C, and E. On the other hand, there was a deficit in iodine and vitamin D intake. In addition, in the case of women, an excess of carbohydrates and sodium intake was observed. In men, we found a deficit in vitamin A and carbohydrate intake. The rest of the average nutrient intakes were the same in both sexes.

### 3.3. Compliance with Recommended Daily Intake of Energy, Vitamins, and Minerals

Regarding the degree of compliance with the recommended daily intakes, Table 3 shows the percentage of compliance of men and women.

Regarding Kcals, statistically significant sex differences were observed, as follows: only 8% (*n =* 16) of the women complied with the recommendations compared with 100% (*n =* 139) of the men.

In the case of vitamins, we highlighted vitamin E, with 16.4% (*n =* 33) of women complying with the recommendations versus 23.7% (*n =* 33) of men; vitamin B1, with 22.4% (*n =* 45) of women versus 41% (*n =* 57) of men; and vitamin B2, with 4% (*n =* 8) of women versus 16.5% (*n =* 32) of men, with statistically significant differences in all three vitamins. Finally, it is noteworthy that in the case of vitamin D and vitamin B12, no women or men met the recommended daily intakes.

In the case of minerals, we noted iron, with a compliance of 15.9% of women compared with 26.6% of men; sodium, with the compliance of 50.2% of women versus 69.1% of men; and iodine, with 31.8% of women compared with 2.2 of men, with statistically significant differences in all three cases (*p* < 0.05).

### 3.4. Correlation between Compliance with Recommended Daily Intakes and Sociodemographic Variables

Table 4 and Table 5 present the relationship between the sociodemographic variables and compliance with the recommended daily intakes. Statistically significant differences in compliance with the recommended daily cholesterol intake were observed according to the area of residence, as follows: 53% of the participants living in urban areas met the recommendations versus 42.9% of individuals living in rural areas. Statistically significant differences were also observed in the compliance with the recommended daily intakes of Kcal and vitamin B1 and its relationship with the level of studies, and, in particular, between the levels of illiteracy and middle studies (Table 4). With regard to compliance with the recommended daily intakes of potassium, statistically significant differences were observed depending on the area of residence as follows: 36.3% of the individuals residing in urban areas complied with the recommendations compared with 25.7% of compliance in residents of rural areas. In the case of fiber, there was a difference according to marital status, in particular between married and divorced people, as follows: 22.2% of the married individuals met the recommended daily intake of fiber versus 17.6% of the divorced people (Table 5). 

### 3.5. Correlation between Compliance with Recommended Daily Intakes and Clinical Variables

Table 6 and Table 7 present the relationship between the clinical variables and compliance with the recommended intakes. Significant differences were observed between systolic blood pressure levels and compliance with the recommended cholesterol intake (systolic blood pressure > 130 mmHg = 41% compliance versus 51.5% with blood pressure < 130 mmHg), compliance with vitamin C intake (systolic blood pressure > 130 mmHg = 54.7% compliance versus 38.4% with blood pressure < 130 mmHg), compliance with vitamin B1 intake (systolic blood pressure >130 mmHg = 24.3% compliance compared to 36.4% with blood pressure < 130 mmHg), and compliance of calcium intake (systolic blood pressure > 130 mmHg = 43% compliance compared with 31.1% with blood pressure < 130 mmHg). Regarding BMI, significant differences in compliance with vitamin B6 intake were observed between overweight individuals (15.6% compliance) and obese individuals (5.7% compliance). In the case of blood cholesterol levels, differences were observed in compliance of the recommended intake of vitamin C (cholesterol < 200 mm/dL = 64.9% of compliance versus 54.9% with cholesterol > 200 mm/dL) and in compliance with vitamin B2 intake (cholesterol < 200 mm/dL = 10.8% of compliance versus 3.9% with cholesterol > 200 mm/dL). Finally, in the case of the blood glucose levels, differences were observed in compliance of the recommended intake of vitamin E (glucose < 120 mm/dL = 20.8% of compliance versus 8.1% of compliance with glucose > 120 mm/dL) and in compliance with sodium intake (glucose < 120 mm/dL = 59.2% compliance versus 37.8% of compliance with glucose >120 mm/dL).

### 3.6. Correlation between Compliance with Recommended Daily Intakes and Lifestyles

Table 8 and Table 9 depict the relationship of compliance with the recommended daily intakes and lifestyles. In tobacco use, statistically significant differences in Kcal intake were observed between regular smokers (58.3% compliance) and non-smokers (41.3% compliance), and in vitamin B6 intake, between occasional smokers (31.2% compliance) and non-smokers (9.5% compliance). Regarding alcohol consumption, statistically significant differences were observed in compliance with Kcal intake between people who consumed alcohol regularly (73.3% compliance) and people who did not consume alcohol (32.9% compliance); in compliance with vitamin B2 intake, between persons who consumed alcohol regularly (16.7% compliance) and individuals who never drank alcohol (3.9% compliance); in compliance with potassium intake, between people who never smoked (32.9% compliance) and regular smokers (13.3% compliance); and in compliance with iodine intake, between individuals who never smoked (29.6% compliance) and regularly smokers (3.3% compliance).

Finally, with regard to physical activity, statistically significant differences in Kcal intake were observed between people performing between 5–10 h of physical activity (56.9% compliance) and individuals who never engaged in physical activity (36.4% compliance); in cholesterol intake, between individuals who performed between 5–10 h of physical activity per week (36.1% compliance) and those who never engaged in physical activity (57.6% compliance); in compliance with vitamin B2 intake, between people who performed between 5–10 h of physical activity per week (18.1% compliance) and those who never engaged in physical activity (5.1% compliance), and between individuals who performed between 5–10 h of physical activity per week (18.1% compliance) and those who performed between 1–2.5 h per week (3% compliance); and in compliance with iodine intake, between people who performed between 5–10 h of physical activity (6.9% compliance) and those who never engaged in physical activity (29.3% compliance).

## 4. Discussion

Assessment of dietary intake is critical in order to understand the relationship between nutrition and the prevention of age-related diseases, as well as the psychosocial factors that may be related to nutritional risk. For the first time, to our knowledge, this study has analyzed the relationship between compliance with the recommended dietary intakes and clinical conditions, and sociodemographic and lifestyle characteristics in a sample of older people residing in the Spanish Mediterranean. Compliance with generally recommended dietary intakes is low, in some cases by deficiency, such as vitamin D, where none of the participating subjects meet the requirements, and iodine, where the compliance rate does not exceed 20%, or by excess, such as with monounsaturated fatty acids, fiber, iron, the B vitamins, vitamin E, and vitamin C. These results partially coincide with those found in international studies, where a clear deficit in the recommended daily intake of vitamin D [27,28,29] and an excess in vitamin C intake [14,28] are observed. On the other hand, the excess of fiber, iron, vitamin E, the B vitamins, and monounsaturated fatty acids do coincide with the results found in the literature, where deficits in fiber and iron and in the levels of vitamin E and the B vitamins are observed [29]. These differences may be due mainly to the Mediterranean diet model, rich in monounsaturated fatty acids and in vitamin E from extra virgin olive oil, rich in fiber, as well as due to the high intake of fruit and vegetables and a correct proportion of high biological quality protein, of both plant and animal origin [9,10].

On the other hand, there are significant associations between lifestyles, sociodemographic characteristics, and clinical variables, and compliance with the recommended daily intakes. As shown by the results found in international studies, our work concludes that living in rural areas (because there are fewer accessible services), being divorced, and being illiterate negatively influence compliance with the recommended intakes of certain nutrients [6,19,30,31]. With regard to lifestyles, as in numerous research studies, we observed that a greater physical activity improves health and, in our case, there is greater compliance with the recommendations of Kcal, cholesterol, and vitamin B2 intake. This may be because people who are physically more active care more about their diet, and this has an impact on their health [19,32,33]. With regard to alcohol consumption, a higher compliance with recommended dietary intakes of Kcal, vitamin B2, potassium, and iodine is observed when alcohol is consumed regularly. These data may be due to the intake of red wine, typical of the Mediterranean diet, associated with the main daily meals and following the usual recommendations of one glass of wine per day [34].

Finally, with regard to the clinical variables, it is observed that those with better blood pressure, cholesterol, and glucose levels comply, to a higher degree, with the recommended intakes.

This study presents some limitations. First, causality cannot be established, as it is a cross-sectional study. Second, the tools used to quantify food intake, such as the food frequency questionnaires, measure food consumption in the past year and are vulnerable to systematic measurement errors, although the use of validated questionnaires reduces this possible limitation. In addition, a portion-sized photographic food atlas was also used to facilitate the completion of questionnaires and to minimize memory bias. In relation to sample selection, it should be noted that we used a convenience sample. Finally, clinical and health data were self-perceived, which may underestimate the results. However, self-perceived data are probably the most reliable and predictive health measurement, and have demonstrated satisfactory validity and reliability in comparisons of measurements made by professionals who are experts in population studies and within a specific context [35,36,37,38,39,40,41].

In conclusion, this study highlights the importance of accurately knowing dietary intakes among the older population and what factors, such as lifestyles or sociodemographic characteristics, may predispose to a better or worse compliance with the recommendations. The studied population has nutritional deficits in the case of vitamin D, essential for maintaining good bone health, and iodine, important for endocrine–metabolic control. In this sense, an inadequate dietary intake can increase the risk of certain chronic-degenerative diseases and of malnutrition and fragility, more pronounced in this age group. In this sense, new public health policies are needed to identify people at nutritional risk and their predisposing factors, as well as concrete nutritional deficits in order to design specific interventions adapted to older people in order to prevent them.

## Figures and Tables

**Table 1:** Sociodemographic variables and lifestyle. SD—standard deviation

	N (%)
Females	201 (59.1)
Males	139 (40.9)
**Mean Age (SD)**	71 ± 8.59
**Place of residence**	
Rural	70 (20.6)
Urban	270 (79.4)
**Marital status**	
Single	9 (2.6)
Married	212 (62.4)
Living with a partner	13 (3.8)
Widowed	95 (27.9)
Divorced	11 (3.2)
**Educational level**	
Illiterate	9 (2.6)
Can read and write	101 (29.7)
Primary studies	128 (37.6)
Middle studies	102 (30)
**Tobacco use**	
No	264 (77.6)
Yes, occasionally	16 (4.7)
Yes, on a regular basis	60 (17.6)
**Alcohol consumption**	
No	152 (44.7)
Yes, occasionally	158 (46.5)
Yes, on a regular basis	30 (8.8)
**BMI**	
Low weight	3 (0.9)
Normal weight	103 (30.0)
Overweight	147 (43.2)
Obesity	88 (25.9)
**Physical activity hours per week M (SD)**	4.23 ± 5.85

**Table 2:** Average nutrient intake based on gender.

Nutrients	Females			Males		
	Mean (SD)	Recommended Intake	Compliance (%)	Mean (SD)	Recommended Intake	Compliance (%)
Kcal	2522.08 ± 757.94	2300	109.66%	2330.00 ± 613.68	2400	97.08%
Proteins (g)	99.60 ± 27.72	41	242.93%	91.49 ± 25.92	54	169.43%
Lipids (g)	128.37 ± 46.08	76.6	167.58%	125.00 ± 40.28	80	156.25%
SFA (g)	35.60 ± 13.40	20.4	174.51%	32.15 ± 11.86	21.4	150.23%
MUFA (g)	65.70 ± 25.06	33.2	197.89%	68.52 ± 23.62	34.6	128.03%
PUFA(g)	20.19 ± 8.19	12.7	158.98%	16.71 ± 6.54	13.3	125.40%
Cholesterol (mg)	341.29 ± 115.51	300	113.76%	301.64 ± 105.26	300mg	100.55%
Carbohydrate (g)	228.43 ± 76.26	316.25	72.23%	196.03 ± 52.02	330	59.40%
Fiber (g)	51.75 ± 24.97	30	172.5%	51.23 ± 45.86	30	170.77%
K (mg)	3245.03 ± 951.54	3500	92.72%	2990.51 ± 863.19	3500	85.44%
Na (mg)	2660.76 ± 803.05	2000	133.04%	2001.59 ± 807.77	2000	100.08%
Calcium (mg)	1062.1 ± 361.11	1200	88.51%	1011.45 ± 303.98	1200	84.29%
Iron (mg)	22.60 ± 9.29	10	226%	18.86 ± 6.98	10	188.6%
Iodine (μg)	84.63 ± 28.25	110	76.94%	78.99 ± 23.77	140	56.42%
Vit B1(mg)	1.32 ± 0.38	0.8	165%	1.20 ± 0.16	1	120%
Vit B2 (mg)	2.75 ± 1.31	1.1	250%	2.72 ± 1.29	1.4	194.27%
Vit B6 (mg)	2.85 ± 0.91	1.6	178.13%	2.69 ± 0.82	1.8	149.44%
Vit B12(μg)	33.59 ± 17.14	2	1679.5%	27.68 ± 14.78	2	1384%
Vit C (mg)	194.08 ± 83.00	60	323.47%	184.11 ± 75.50	60	306.85%
Vit A(μg)	961.75 ± 374.19	800	120.22%	865.33 ± 291.91	1000	86.53%
Vit D(μg)	5.43 ± 2.43	20	27.15%	4.54 ± 2.13	15	30.27%
Vit E (mg)	17.92 ± 8.24	12	149.33%	15.34 ± 5.08	12	127.83%

SFA—saturated fatty acids; MUFA—monounsaturated fatty acids; PUFA—polyunsaturated fatty acids. The recommended daily intake values of energy and nutrients come from the food composition tables for the Spanish population [26].

**Table 3:** Differences in compliance with recommended daily intakes as a function of sex.

Nutrients	Complies			Does Not Comply		
	Females (%)	Males (%)	Total (%)	Females (%)	Males (%)	Total (%)
Kcal *	16 (8.0)	139 (100.0)	155 (45.5)	185 (92.0)	0 (0.0)	185(54.4)
Cholesterol	105 (52.2)	68 (48.9)	173 (50.9)	96 (47.8)	71 (51.1)	167 (49.1)
Fiber	33 (16.4)	27 (19.4)	60 (17.6)	168 (83.6)	112 (80.6)	280 (82.4)
Iron *	32 (15.9)	37 (26.6)	69 (20.3)	169 (84.1)	102 (73.4)	271 (79.7)
Potassium	71 (35.3)	45 (32.4)	116 (34.1)	130 (64.7)	94 (67.6)	224 (65.7)
Vitamin C	120 (59.7)	92 (66.2)	212 (62.4)	81 (40.3)	47 (33.8)	128 (37.6)
Vitamin E *	33 (16.4)	33 (23.7)	66 (19.4)	168 (83.6)	106 (76.5)	274 (80.6)
Sodium *	101 (50.2)	96 (69.1)	197 (57.9)	100 (49.8)	43 (30.9)	143 (42.1)
Calcium	78 (38.8)	61 (43.9)	139 (40.9)	123 (61.2)	78 (56.1)	201 (59.1)
Vit B12	0 (0.0)	0 (0.0)	0 (0.0)	201 (100.0)	139 (100.0)	340 (100.0)
Vit D	0 (0.0)	0 (0.0)	0 (0.0)	201 (100.0)	139 (100.0)	340 (100.0)
Iodine *	64 (31.8)	3 (2.2)	67 (19.7)	137 (68.2)	136 (97.8)	273 (80.3)
Vit B1 *	45 (22.4)	57 (41.0)	102 (30.0)	156 (77.6)	82 (59.0)	238 (70.0)
Vit B	8 (4.0)	23 (16.5)	31 (9.1)	193 (96.0)	116 (83.5)	309 (90.9)
Vit B6	19 (9.5)	21 (15.1)	40 (11.8)	182 (90.5)	118 (84.9)	300 (88.2)
Protein	0 (0.0)	2 (1.4)	2 (0.6)	201 (100.0)	137 (98.6)	338 (99.4)

The recommended daily intake values of energy and nutrients come from the food composition tables for the Spanish population [26]; * Statistically significant differences between groups (*p* < 0.05).

**Table 4:** Relationship between sociodemographic variables and compliance with daily intake recommendations for vitamins, energy, and cholesterol.

	Kcal		Chol		Vit C		Vit E		Vit B1		Vit B2		Vit B6		Vit B12		Vit D	
	C	NC	C	NC	C	NC	C	NC	C	NC	C	NC	C	NC	C	NC	C	NC
	N (%)	N (%)	N (%)	N (%)	N (%)	N (%)	N (%)	N (%)	N (%)	N (%)	N (%)	N (%)	N (%)	N (%)	N (%)	N (%)	N (%)	N (%)
**Place of residence**																		
Rural	33 (47.1)	37 (52.9)	30 (42.9)	40 (57.1) ^a^	44 (62.9)	26 (37.1)	11 (15.7)	59 (84.3)	23 (32.9)	47 (67.1)	6 (8.6)	64 (91.4)	9 (12.9)	61 (87.1)	0 (0.0)	70 (100.0)	0 (0.0)	70 (100.0)
Urban	122 (45.2)	148 (54.8)	143 (53.0)	127 (47.0)	168 (62.2)	102 (37.8)	55 (20.4)	215 (79.6)	79 (29.3)	191 (70.7)	25 (9.3)	245 (90.7)	31 (11.5)	239 (88.5)	0 (0.0)	270 (100.0)	0 (0.0)	270 (100.0)
**Marital status**																		
Single	3 (3.3)	6 (66.7)	5 (55.6)	4 (44.4)	5 (55.6)	4 (44.4)	1 (11.1)	8 (88.9)	4 (44.4)	5 (55.6)	0 (0.0)	9 (100.0)	1 (11.1)	8 (88.9)	0 (0.0)	9 (100.0)	0 (0.0)	9 (100.0)
Married	104 (49.1)	108 (50.9)	105 (49.5)	107 (50.5)	139 (65.6)	73 (34.4)	41 (19.3)	171 (80.7)	71 (33.5)	141 (66.5)	25 (11.8)	187 (88.2)	25 (11.8)	187 (88.2)	0 (0.0)	212 (100.0)	0 (0.0)	212 (100.0)
Lives with a partner	8 (61.5)	5 (38.5)	8 (61.5)	5 (38.5)	9 (69.2)	4 (30.8)	1 (7.7)	12 (92.3)	3 (23.1)	10 (76.9)	1 (7.7)	12 (92.3)	3 (23.1)	10 (76.9)	0 (0.0)	13 (100.0)	0 (0.0)	13 (100.0)
Widowed	36 (37.9)	59 (62.1)	48 (50.5)	37 (49.5)	52 (54.7)	43 (45.3)	20 (21.1)	75 (78.9)	21 (22.1)	74 (77.9)	5 (5.3)	90 (94.7)	11 (11.6)	84 (88.4)	0 (0.0)	95 (100.0)	0 (0.0)	95 (100.0)
Divorced	4 (36.4)	7 (63.6)	7 (36.4)	4 (36.4)	7 (63.6)	4 (36.4)	3 (27.3)	8 (72.7)	3 (27.3)	8 (72.7)	0 (0.0)	11 (100.0)	0 (0.0)	11 (100.0)	0 (0.0)	11 (100.0)	0 (0.0)	11 (100.0)
**Educational level**																		
Illiterate	1 (11.1)	8 (88.9) ^b^	3 (33.3)	6 (66.7)	5 (55.6)	4 (44.4)	2 (22.2)	7 (77.8)	0(100.0)	9 (100.0) ^b^	1 (11.1)	8 (88.9)	1 (11.1)	8 (88.9)	0 (0.0)	9 (100.0)	0 (0.0)	9.0 (100.0)
Can read and write	46 (45.5)	55 (54.5)	56 (55.4)	45 (44.6)	66 (65.3)	35 (34.7)	16 (15.8)	85 (84.2)	25 (24.8)	76 (75.2)	13 (12.9)	88 (91.1)	13 (12.9)	88 (87.1)	0 (0.0)	101 (100.0)	0 (0.0)	101 (100.0)
Primary Studies	51 (39.8)	77 (60.2)	66 (51.6)	62 (48.4)	86 (67.2)	42 (32.8)	26 (20.3)	102 (79.7)	38 (29.7)	90 (70.3)	10 (7.8)	118 (92.2)	15 (11.7)	113 (88.3)	0 (0.0)	128 (100.0)	0 (0.0)	128 (100.0)
Middle studies	57 (55.9)	45 (44.1)	48 (47.1)	54 (52.9)	55 (53.9)	47 (46.1)	22 (21.6)	80 (78.4)	39 (38.2)	63 (61.8)	11 (10.8)	91 (89.2)	11 (10.8)	91 (89.2)	0 (0.0)	102 (100.0)	0 (0.0)	102 (100.0)
**Age**																		
60–70	88 (48.9)	92 (51.1)	85 (47.2)	95 (52.8)	124 (68.9)	56 (31.1) ^c^	43 (23.9)	137 (76.1)	58 (32.2)	122 (67.8)	18 (10.0)	162 (90.0)	20 (11.1)	160 (88.9)	0 (0.0)	180 (100.0)	0 (0.0)	180 (100.0)
71–80	47 (46.5)	54 (53.5)	57 (56.4)	44 (43.6)	53 (52.5)	48 (47.5)	15 (14.9)	86 (85.1)	31 (30.7)	70 (69.3)	9 (8.9)	92 (91.1)	12 (11.9)	89 (88.1)	0 (0.0)	101 (100.0)	0 (0.0)	101 (100.0)
>80	20 (33.9)	39 (66.1)	31 (52.5)	28 (47.5)	35 (59.3)	24 (40.7)	8 (13.6)	51 (86.4)	13 (22.0)	46 (78.0)	4 (6.8)	55 (93.2)	8 (13.6)	51 (86.4)	0(0.0)	59 (100.0)	0 (0.0)	59 (100.0)

Chol—cholesterol; C—complies with the recommendations; NC—does not comply with the recommendations; h—hours; significant differences (*p* < 0.05) between groups (groups without significant differences are not indicated): ^a^ rural and urban; ^b^ illiterate and middle studies; ^c^ 60–70 years and 71–80 years.

**Table 5:** Relationship between sociodemographic variables and compliance with mineral and fiber intake recommendations.

	Fe		K		Na		Ca		Iodine		Fiber	
	C	NC	C	NC	C	NC	C	NC	C	NC	C	NC
**Place of residence**												
Rural	14 (20.0)	56 (80.0)	18 (25.7)	52 (74.0) ^a^	37 (52.9)	33 (47.1)	27 (38.6)	43 (61.4)	16 (22.9)	54 (77.1)	11 (15.7)	59 (84.3)
Urban	55 (20.4)	215 (79.6)	98 (36.3)	172 (63.7)	160 (59.3)	110 (40.7)	112 (41.5)	158 (58.5)	51 (18.9)	219 (81.1)	49 (18.1)	221 (81.9)
**Marital status**												
Single	3 (33.3)	6 (66.7)	5 (55.6)	4 (44.4)	4 (44.4)	5 (55.6)	5 (55.6)	4 (44.4)	3 (33.3)	6 (66.7)	1 (11.1)	8 (88.9)
Married	48 (22.6)	164 (77.4)	7 (33.0)	142 (67.0)	133 (62.7)	79 (37.3)	89 (42.0)	123 (58.0)	39 (18.4)	173 (81.6)	47 (22.2)	165 (77.8) ^b^
Living with a partner	3 (23.1)	10 (76.9)	3 (30.8)	9 (69.2)	9 (69.2)	4 (30.8)	4 (30.8)	9 (69.2)	1 (7.7)	12 (92.3)	0 (0.0)	13 (100.0)
Widowed	14 (14.7)	81 (85.3)	36 (37.9)	59 (62.1)	46 (48.4)	49 (51.6)	38 (40.0)	57 (60.0)	22 (23.2)	73 (76.8)	11 (11.6)	84 (88.4)
Divorced	1 (9.1)	10 (90.9)	1 (9.1)	10 (90.9)	5 (45.5)	6 (54.5)	3 (27.2)	8 (72.7)	2 (19.7)	273 (80.2)	1 (17.6)	10 (90.9)
**Educational level**												
Illiterate	1 (11.1)	8 (88.9)	1 (11.1)	8 (88.9)	4 (44.4)	5 (55.6)	4 (44.4)	5 (55.6)	4 (44.4)	5 (55.6)	3 (33.3)	6 (66.7)
Can read and write	22 (21.8)	79 (78.2)	35 (34.7)	66 (65.3)	57 (56.4)	44 (43.6)	40 (39.6)	61 (60.4)	21 (20.8)	80 (79.2)	16 (15.8)	85 (84.2)
Primary studies	24 (18.8)	104 (81.2)	42 (37.3)	86 (67.2)	70 (54.7)	58 (45.3)	56 (43.8)	72 (56.3)	30 (23.4)	98 (76.6)	23 (18.0)	105 (82.0)
Secondary studies	22 (21.6)	80 (78.4)	38 (37.2)	64 (62.7)	66 (64.7)	36 (35.3)	39 (38.2)	63 (61.8)	12 (11.8)	90 (88.2)	18 (17.6)	84 (82.4)
**Age (years)**												
60–70	40 (22.2)	140 (77.8)	61 (33.9)	119 (66.1)	106 (58.9)	74 (41.1)	75 (41.7)	105 (58.3)	34 (18.9)	146 (81.8)	37 (20.6)	143 (79.4)
70–80	18 (17.8)	83 (82.2)	34 (33.7)	67 (66.3)	58 (57.4)	43 (42.6)	42 (41.6)	59 (58.4)	18 (17.8)	83 (82.2)	17 (16.8)	84 (83.2)
>80	11 (18.6)	48 (81.4)	21 (35.6)	38 (64.4)	33 (55.9)	26 (44.1)	22 (37.3)	37 (62.7)	15 (25.4)	44 (74.6)	6 (10.2)	53 (89.8)

Fe—iron; K—potassium; Na—sodium; Ca—calcium; C—complies; NC—does not comply; significant differences (*p* < 0.05) between groups (groups without significant differences are not indicated): ^a^ rural and urban; ^b^ married and divorced.

**Table 6:** Relationship between sociodemographic variables and compliance with daily intake recommendations for vitamins, energy, and cholesterol.

	Kcal		Col		Vit C		Vit E		Vit B1		Vit B2		Vit B6		Vit B12		Vit D	
	C	NC	C	NC	C	NC	C	NC	C	NC	C	NC	C	NC	C	NC	C	NC
**Systolic blood pressure**																		
>130 mmHg	88 (41.1)	126 (58.9) ^a^	97 (45.3)	117 (54.7) ^a^	123 (57.5)	91 (42.5) ^a^	40 (18.7)	174 (81.3)	52 (24.3)	162 (75.7) ^a^	21 (9.8)	193 (90.2)	25 (11.7)	189 (88.3)	0 (0.0)	214 (100.0)	0 (0.0)	214 (100.0)
<130 mmHg	51 (51.5)	48 (48.5)	61 (61.6)	38 (38.4)	69 (69.7)	30 (30.3)	22 (22.2)	77 (77.8)	36 (36.4)	63 (63.6)	6 (6.1)	93 (93.9)	11 (11.1)	88 (88.0)	0 (0.0)	99 (100.0)	0 (0.0)	99 (100.0)
**Diastolic blood pressure**																		
<100 mmHg	107 (42.6)	144 (57.4)	123 (49.0)	128 (51.0)	152 (60.6)	99 (39.4)	47 (18.7)	204 (81.3)	70 (27.9)	181 (72.1)	22 (8.8)	229 (91.2)	32 (12.7)	219 (87.3)	0 (0.0)	251 (100.0)	0 (0.0)	251 (100.0)
>100 mmHg	33 (50.0)	33 (50.0)	38 (57.6)	28 (42.4)	41 (62.1)	25 (37.9)	14 (21.2)	52 (78.8)	20 (30.3)	46 (69.7)	5 (7.6)	61 (92.4)	4 (6.1)	62 (93.9)	0 (0.0)	66 (100.0)	0 (0.0)	66 (100.0)
**BMI**																		
Low weight	2 (66.7)	1 (33.3)	2 (66.6)	1 (33.3)	2 (66.6)	1 (33.3)	0 (0.0)	3 (100.0)	1 (33.3)	2 (66.6)	0 (0.0)	3 (100.0)	1 (33.3)	2 (66.6)	0 (0.0)	3 (100.0)	0 (0.0)	3(100.0)
Normal weight	48 (47.1)	54 (52.9)	51 (50.0)	51 (50.0)	65 (63.7)	37 (36.3)	14 (13.7)	88 (86.3)	31 (30.4)	71 (69.6)	9 (8.8)	93 (91.2)	11 (10.8)	91 (89.2)	0 (0.0)	102 (100.0)	0 (0.0)	102 (100.0)
Overweight	70 (47.6)	77 (52.4)	79 (53.7)	68 (46.3)	88 (59.9)	59 (40.1)	37 (25.2)	110 (74.8)	42 (28.6)	105 (71.4)	14 (9.5)	133 (90.5)	23 (15.6)	124 (84.4) ^b^	0 (0.0)	147 (100.0)	0 (0.0)	147 (100.0)
Obese	35 (39.8)	53 (60.2)	41 (46.6)	47 (53.4)	57 (64.8)	31 (35.2)	15 (17.0)	73 (83.0)	28 (31.8)	60 (68.2)	8 (9.1)	80 (90.9)	5 (5.7)	83 (94.3)	0 (0.0)	88 (100.0)	0 (0.0)	88 (100.0)
**Cholesterol**																		
<200 mm/dL	90 (46.4)	104 (53.6)	94 (48.5)	100 (51.5)	126 (64.9)	68 (35.1) ^c^	40 (20.6)	154 (79.4)	59 (30.4)	135 (69.5)	21 (10.8)	173 (89.2) ^c^	27 (13.9)	167 (86.1)	0 (0.0)	158 (100.0)	0 (0.0)	158 (100.0)
>200 mm/dL	42 (42.2)	59 (57.8)	58 (56.9)	44 (43.1)	56 (54.9)	46 (45.1)	17 (16.7)	85 (83.3)	29 (28.4)	73 (71.6)	4 (3.9)	98 (96.1)	8 (7.8)	94 (92.2)	0 (0.0)	30 (100.0)	0 (0.0)	30 (100.0)
**Glucose**																		
<120 mm/dL	111 (42.7)	149 (57.3)	133 (51.2)	127 (48.8)	158 (60.8)	102 (39.2)	54 (20.8)	206 (79.2) ^d^	73 (28.1)	187 (71.9)	24 (9.2)	236 (90.8)	32 (12.3)	228 (87.7)	0 (0.0)	194 (100.0)	0 (0.0)	194 (100.0)
>120 mm/dL	20 (54.1)	17 (45.9)	19 (51.4)	18 (48.6)	26 (70.3)	11 (29.7)	3 (8.1)	34 (91.9)	13 (35.2)	24 (64.9)	1 (8.4)	36 (91.6)	4 (10.8)	33 (89.2)	0 (0.0)	102 (100.0)	0 (0.0)	102 (100.0)

C—complies with the recommendations; NC—does not comply with the recommendations; BMI—body mass index; significant differences (*p* < 0.05) between groups (the groups with no significant differences are not indicated), ^a^ Systolic tension >130 mmHg and systolic tension <130 mHg; ^b^ overweight and obese; ^c^ total cholesterol <200 and total cholesterol >200; ^d^ glucose levels <120 and glucose levels >120.

**Table 7:** Relationship between lifestyles and compliance with daily recommended intakes for minerals and fiber.

	Fe		K		Na		Ca		Iodine		Fiber	
	C	NC	C	NC	C	NC	C	NC	C	NC	C	NC
**Tobacco use**												
Never	54 (20.5)	210 (79.5)	91 (34.5)	173 (65.5)	153 (58)	111 (42.0)	102 (38.6)	162 (61.4)	55 (20.8)	209 (79.2)	46 (17.4)	218 (82.6)
Yes, sporadic	3 (18.8)	12 (81.2)	5 (31.2)	11 (68.8)	9 (56.2)	7 (43.8)	7 (43.8)	9 (56.2)	2 (12.5)	14 (87.5)	4 (25.0)	12 (75.0)
Yes, habitual	12 (20.0)	48 (80.0)	20 (34.10)	40 (65.0)	35 (58.3)	25 (41.7)	30 (50.0)	30 (50.0)	10 (16.7)	50 (82.2)	10 (16.7)	50 (83.3)
**Alcohol consumption**												
Never	29 (19.2)	123 (80.9)	50 (32.9)	102 (67.1) ^a^	80 (52.6)	72 (47.4)	59 (38.8)	93 (61.2)	45 (29.6)	107 (70.4) ^a^	21 (13.8)	131 (86.2)
Yes, sporadic	31 (19.6)	127 (80.4)	62 (31.8)	96 (60.8)	96 (60.8)	62 (39.2)	66 (41.8)	92 (58.2)	21 (13.3)	137 (86.7)	34 (21.5)	127 (80.4)
Yes, habitual	9 (30.0)	21 (70.0)	4 (13.3)	26 (86.7)	21 (70.0)	9 (30.0)	14 (46.7)	16 (53.3)	1 (3.3)	29 (96.7)	5 (16.7)	25 (83.3)
**Physical activity**												
0 h	14 (14.1)	85 (85.9)	31 (31.3)	68 (68.7)	55 (55.6)	44 (44.4)	50 (50.5)	49 (49.5)	29 (29.3)	70 (70.7) ^b^	16 (16.2)	83 (83.8)
1–2.5 h	11 (16.7)	55 (78.8)	32 (48.5)	34 (51.5)	32 (48.5)	34 (51.5)	24 (36.4)	42 (63.6)	14 (21.2)	52 (78.8)	14 (21.1)	52 (78.8)
2.5–5 h	14 (19.7)	57 (80.3)	21 (29.6)	50 (70.4)	43 (66.7)	28 (39.2)	28 (39.4)	43 (60.6)	13 (18.3)	58 (81.7)	9 (12.7)	62 (87.3)
5–10 h	20 (27.8)	52 (72.2)	21 (29.2)	51 (70.8)	48 (66.7)	24 (33.3)	26 (36.1)	46 (63.9)	5 (6.9)	67 (93.1)	15 (20.8)	57 (79.2)
>10 h	7 (28.0)	18 (72.0)	10 (40.0)	15 (60.0)	14 (56.0)	11 (44.0)	9 (36.0)	16 (64.0)	6 (24.0)	19 (76.0)	6 (24.0)	19 (76.0)

Fe—iron; K—potassium; Na—sodium; Ca—calcium; C—complies; NC—does not comply; significant differences (*p* < 0.05) between groups (the groups with no significant differences are not indicated). ^a^ Never drinks alcohol and drinks on a regular basis; ^b^ 0 h of physical activity per week and 5–10 h of physical activity a week.

**Table 8:** Relationship between lifestyle variables and compliance with daily intake recommendations for vitamins, energy, and cholesterol.

	Kcal		Chol		Vit C		Vit E		Vit B1		Vit B2		Vit B6		Vit B12		Vit D	
	C	NC	C	NC	C	NC	C	NC	C	NC	C	NC	C	NC	C	NC	C	NC
	N (%)	N (%)	N (%)	N (%)	N (%)	N (%)	N (%)	N (%)	N (%)	N (%)	N (%)	N (%)	N (%)	N (%)	N (%)	N (%)	N (%)	N (%)
**Tobacco consumption**																		
No	109 (41.3)	155 (58.7) ^a^	137 (51.9)	127 (48.1)	165 (62.5)	99 (37.5)	49 (18.6)	215 (81.4)	75 (28.4)	189 (71.6)	22 (8.3)	242 (91.7)	25 (9.5)	239 (90.5) ^b^	0 (0.0)	264 (100.0)	0 (0.0)	264 (100.0)
Yes, occasional	11 (68.8)	5 (31.2)	4 (25.0)	12 (75.0)	11 (68.8)	5 (31.2)	5 (31.2)	11 (68.8)	4 (25.0)	12 (75.0)	3 (18.8)	13 (81.2)	5 (31.2)	11 (68.8)	0 (0.0)	16 (100.0)	0 (0.0)	16 (100.0)
Yes, habitual	35 (58.3)	25 (41.7)	32 (53.3)	28 (46.7)	36 (60.0)	24 (40.0)	12 (20.0)	48 (80.0)	23 (38.3)	37 (61.7)	6 (10.0)	54 (90.0)	10 (1.7)	50 (83.3)	0 (0.0)	60 (100.0)	0 (0.0)	60 (100.0)
**Alcohol consumption**																		
Never	50 (32.9)	102 (67.1)c	71 (46.7)	81 (53.3)	90 (59.2)	62 (40.8)	31 (20.4)	121 (79.6)	39 (25.7)	113 (74.3)	6 (3.9)	146 (96.1) ^c^	17 (11.2)	135 (88.8)	0 (0.0)	152 (100.0)	0 (0.0)	152 (100.0)
Yes, occasional	83 (52.5)	75 (47.5)	86 (54.4)	72 (45.2)	100 (63.3)	58 (36.7)	31 (19.6)	127 (80.4)	53 (33.5)	105 (66.5)	20 (16.7)	138 (87.3)	19 (12.0)	139 (88.0)	0 (0.0)	158 (100.0)	0 (0.0)	158 (100.0)
Yes, habitual	22 (73.3)	8 (26.7)	16 (53.3)	14 (46.7)	22 (73.3)	8 (26.7)	4 (13.3)	26 (86.7)	10 (33.3)	20 (66.7)	5 (16.7)	25 (83.3)	4 (13.3)	26 (86.8)	0 (0.0)	30 (100.0)	0 (0.0)	30 (100.0)
**Physical activity**																		
0 h	36 (36.4)	63 (63.6) ^d^	42 (42.4)	57 (57.6) ^d^	54 (54.5)	45 (45.5)	16 (16.2)	83 (83.8)	25 (25.3)	74 (74.7)	5 (5.1)	94 (94.9) ^d,e^	9 (9.1)	90 (90.9)	0 (0.0)	99 (100.0)	0 (0.0)	9 (100.0)
1–2.5 h	32 (48.5)	34 (63.6)	36 (54.5)	30 (45.5)	38 (57.6)	28 (42.4)	11 (16.7)	55 (83.3)	17 (25.8)	49 (74.2)	2 (3.0)	64 (97.0)	6 (9.1)	60 (90.9)	0 (0.0)	66 (100.0)	0 (0.0)	66 (100.0)
2.5–5 h	26 (36.6)	45 (63.4)	35 (49.3)	36 (50.7)	46 (64.8)	25 (35.2)	12 (16.9)	59 (83.1)	23 (32.4)	48 (67.6)	6 (8.5)	65 (91.5)	8 (11.3)	63 (88.7)	0 (0.0)	71 (100.0)	0 (0.0)	71 (100.0)
5–10 h	41 (56.9)	31 (43.1)	46 (63.9)	26 (36.1)	52 (72.2)	20 (27.8)	18 (25.0)	54 (75.0)	28 (38.9)	44 (61.1)	13 (18.1)	59 (81.9)	9 (12.5)	63 (87.5)	0 (0.0)	72 (100.0)	0 (0.0)	72 (100.0)
>10 h	15 (60.0)	10 (40.0)	12 (48.0)	13 (52.0)	17 (68.0)	8 (32.0)	7 (28.0)	18 (72.0)	5 (20.0)	20 (80.0)	2 (8.0)	23 (92.0)	4 (16.0)	21 (84.0)	0 (0.0)	25 (100.0)	0 (0.0)	25 (100.0)

Col—cholesterol; C—complies with the recommendations; NC—does not comply with the recommendations; h—hours; significant differences (*p* < 0.05) between groups (groups without significant differences are not indicated): ^a^ non-smoker and habitual smoker; ^b^ non-smoker and occasional smoker; ^c^ never drinks alcohol and drinks habitually; ^d^ 0 h of physical activity per week and 5–10 h of physical activity per week; ^e^ 1–2.5 h of physical activity per week and 5–10 h of physical activity per week.

**Table 9:** Relationship between clinical variables and compliance with recommendations for mineral and fiber intake.

	Fe		K		Na		Ca		Iodine		Fiber	
	C	NC	C	NC	C	NC	C	NC	C	NC	C	NC
**Systolic blood pressure**												
>130 mmHg	42 (19.6)	172 (80.4)	72 (33.6)	142 (66.4)	122 (57.0)	92 (43.0)	92 (43.0)	122 (57.0) ^a^	45 (21.0)	169 (79.0)	35 (16.4)	179 (83.6)
<130 mmHg	17 (17.2)	82 (82.8)	36 (36.4)	63 (63.6)	55 (55.6)	44 (44.4)	31 (31.1)	68 (68.7)	16 (16.2)	83 (83.8)	18 (18.2)	81 (81.8)
**Diastolic blood pressure**												
<100 mmHg	50 (19.9)	201 (80.1)	91 (36.3)	160 (63.7)	146 (58.2)	105 (41.8)	105 (41.8)	146 (58.2)	51 (20.3)	200 (79.7)	44 (17.5)	207 (82.5)
>100 mmHg	56 (84.8)	17 (25.8)	17 (25.8)	49 (74.2)	36 (54.5)	30 (45.5)	22 (33.3)	44 (66.7)	13 (19.7)	53 (80.3)	9 (13.6)	57 (86.4)
**BMI**												
Low weight	0 (0.0)	3 (100.0)	2 (66.6)	1 (33.3)	1 (33.3)	2 (66.6)	1 (33.3)	2 (66.6)	1 (33.3)	2 (66.6)	0 (0.0)	3 (100.0)
Normal weight	26 (25.5)	76 (74.5)	28 (27.5)	74 (72.5)	65 (63.7)	37 (36.3)	42 (41.2)	60 (58.8)	24 (23.5)	78 (76.5)	25 (24.5)	77 (75.5)
Overweight	25 (17.0)	122 (83.0)	55 (37.4)	92 (62.6)	82 (55.8)	65 (44.2)	60 (40.8)	87 (59.2)	22 (15.0)	125 (85.0)	23 (15.6)	124 (84.4)
Obesity	18 (20.5)	70 (79.5)	31 (35.2)	57 (64.8)	49 (55.7)	39 (44.3)	36 (40.9)	52 (59.1)	20 (22.7)	68 (77.3)	12 (13.6)	76 (86.4)
**Cholesterol**												
<200 mg/dL	40 (20.6)	154 (79.4)	64 (33.0)	130 (67.0)	113 (58.2)	81 (41.8)	76 (39.2)	118 (60.8)	3 (33.3)	6 (66.7)	36 (18.6)	158 (18.6)
>200 mg/dL	17 (16.7)	85 (83.3)	38 (37.3)	64 (62.7)	55 (53.9)	47 (46.1)	42 (41.2)	60 (58.8)	39 (18.4)	173 (81.6)	13 (12.7)	89 (87.3)
**Glucose levels**												
<120 mm/dL	54 (20.8)	206 (79.2)	87 (33.5)	173 (66.5)	154 (59.2)	106 (40.8) ^b^	104 (40.0)	156 (60.0)	38 (19.6)	156 (80.4)	43 (16.5)	217 (83.5)
>120 mm/dL	4 (10.8)	33 (89.2)	14 (37.8)	23 (62.2)	14 (37.8)	23 (62.2)	17 (45.9)	20 (54.1)	19 (18.6)	83 (81.4)	6 (16.2)	31 (83.8)

C—complies with the recommendations; NC—does not comply with the recommendations; BMI—body mass index; significant differences *p* < 0.05 between groups (groups without significant differences are not indicated): ^a^ systolic blood pressure <130 mmHg and systolic blood pressure >130 mmHg; ^b^ Glucose levels <120 and glucose levels >120.
